# Development of Receptor Binding Domain (RBD)‐Conjugated Nanoparticle Vaccines with Broad Neutralization against SARS‐CoV‐2 Delta and Other Variants

**DOI:** 10.1002/advs.202105378

**Published:** 2022-02-10

**Authors:** Ran Chen, Xiantao Zhang, Yaochang Yuan, Xiaohui Deng, Bolin Wu, Zhihui Xi, Guanwen Wang, Yingtong Lin, Rong Li, Xuemei Wang, Fan Zou, Liting Liang, Haiping Yan, Chaofeng Liang, Yuzhuang Li, Shijian Wu, Jieyi Deng, Mo Zhou, Xu Zhang, Congrong Li, Xiuqing Bu, Yi Peng, Changwen Ke, Kai Deng, Xin He, Yiwen Zhang, Zhenhai Zhang, Ting Pan, Hui Zhang

**Affiliations:** ^1^ Institute of Human Virology Key Laboratory of Tropical Disease Control of Ministry Education Guangdong Engineering Research Center for Antimicrobial Agent and Immunotechnology Zhongshan School of Medicine Sun Yat‐sen University Guangzhou Guangdong 510080 China; ^2^ Center for Infection and Immunity Study School of Medicine Shenzhen Campus of Sun Yat‐sen University Shenzhen Guangdong 518107 China; ^3^ Guangdong Provincial People's Hospital Guangdong Academy of Medical Sciences Guangzhou Guangdong 510080 China; ^4^ Qianyang Biomedical Research Institute Guangzhou Guangdong 510063 China; ^5^ Department of Gastroenterology The Eight Affiliated Hospital Sun Yat‐sen University Shenzhen Guangdong 518033 China; ^6^ Guangzhou National Laboratory Bio‐Island Guangzhou Guangdong 510320 China; ^7^ BSL‐3 Laboratory Zhongshan School of Medicine Sun Yat‐sen University Guangzhou Guangdong 510080 China; ^8^ Guangdong Provincial Center for Disease Control and Prevention Guangzhou 511430 China; ^9^ Department of Immunology Zhongshan School of Medicine Sun Yat‐sen University Guangzhou 510080 China; ^10^ Center for Precision Medicine Guangdong Provincial People's Hospital Guangdong Academy of Medical Sciences Guangzhou 510080 China

**Keywords:** Delta variants, nanoparticle vaccines, SARS‐CoV‐2

## Abstract

The SARS‐CoV‐2 Delta (B.1.617.2) strain is a variant of concern (VOC) that has become the dominant strain worldwide in 2021. Its transmission capacity is approximately twice that of the original strain, with a shorter incubation period and higher viral load during infection. Importantly, the breakthrough infections of the Delta variant have continued to emerge in the first‐generation vaccine recipients. There is thus an urgent need to develop a novel vaccine with SARS‐CoV‐2 variants as the major target. Here, receptor binding domain (RBD)‐conjugated nanoparticle vaccines targeting the Delta variant, as well as the early and Beta/Gamma strains, are developed. Under both a single‐dose and a prime‐boost strategy, these RBD‐conjugated nanoparticle vaccines induce the abundant neutralizing antibodies (NAbs) and significantly protect hACE2 mice from infection by the authentic SARS‐CoV‐2 Delta strain, as well as the early and Beta strains. Furthermore, the elicitation of the robust production of broader cross‐protective NAbs against almost all the notable SARS‐CoV‐2 variants including the Omicron variant in rhesus macaques by the third re‐boost with trivalent vaccines is found. These results suggest that RBD‐based monovalent or multivalent nanoparticle vaccines provide a promising second‐generation vaccine strategy for SARS‐CoV‐2 variants.

## Graphical abstract

Receptor binding domain (RBD)‐conjugated nanoparticle vaccines are developed with broad neutralization against SARS‐CoV‐2 Delta and other variants. There is a robust production of broader cross‐protective NAbs against almost all the notable SARS‐CoV‐2 variants including the Omicron in rhesus macaques by the third re‐boost with trivalent vaccines. These multivalent nanoparticle vaccines might be a promising second‐generation vaccine for SARS‐CoV‐2 variants.

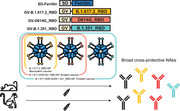

## 1 Introduction

Severe acute respiratory syndrome coronavirus type 2 (SARS‐CoV‐2) is a coronavirus that has caused the epidemic of the respiratory infection coronavirus disease 2019 (COVID‐19). Over the past two years, this virus has rapidly spread across the world, causing a global pandemic.^[^ ^1^ ^]^ Although vaccination has been extensively used worldwide, people still live under the threat of COVID‐19.^[^ ^2^ ^]^ Within the last two years, researchers worldwide recorded the emergence of polymorphisms in the coding sequences across the SARS‐CoV‐2 genome.^[^ ^3^ ^]^ Some of these genomic mutations affect the efficiency of virus transmission, sensitivity to vaccines, and pathogenicity to human beings, and are called variants of concern (VOCs). The emergence of SARS‐CoV‐2 variants has brought greater challenges to the development of vaccines and therapeutic drugs. To date, five VOCs, including the Alpha variant (B.1.1.7),^[^ ^4^ ^]^ Beta variant (B.1.351),^[^ ^5^ ^]^ Gamma variant (P.1),^[^ ^6^ ^]^ Delta variant (B.1.617.2) ^[^ ^7^ ^]^ and the Omicron variant (B.1.1.529) ^[^ ^8^ ^]^ have been reported.^[^ ^9^ ^]^ Compared with the early strain (Wuhan‐Hu‐1), all VOCs carry mutations in the receptor binding domain (RBD). The N501Y mutation located on the RBD is common to all VOCs except the Delta variant. In addition, the key mutations (K417T/N, E484K, N501Y) of the RBD region in the Beta/Gamma variants lead to significant immune evasion, which could reduce the effectiveness of some antibody therapies, and weaken the protection of the vaccine.^[^ ^10^ ^]^ However, the infection rate of these variants, as well as the early strain, have recently declined, and now account for a relatively low proportion of global infections. Conversely, the Delta strain of SARS‐CoV‐2 was first identified in September 2020 and harbors two mutations in the RBD of the S protein, including L452R and T478K (https://www.gisaid.org/hcov19‐variants/).^[^ ^11^ ^]^ This variant has a high transmissibility and has been shown to cause the breakthrough infection in some vaccinated people.^[^ ^12^ ^]^ Consequently, before the emergence of the Omicron variant (B.1.1.529), the Delta variant was the most transmissible and virulent strain among all the variants in 2021. However, the Omicron strain of SARS‐CoV‐2, first reported in November 2021, carries as much as 15 mutations in the RBD of the S protein, including mutations of Beta (K417N, E484K, and N501Y) and the mutation of Delta (T478K). It is defined as the fifth VOC due to its multiple mutations that may have an impact on transmissibility and immune evasion. As such, the strategies to fight the COVID‐19 pandemic, either by vaccines or therapeutic interventions, have been threatened by the emergence of the Delta variant and other SARS‐CoV‐2 variants.

At present, mRNA vaccines, inactivated vaccines, and recombinant protein‐based vaccines have been approved as prophylactic efforts to prevent COVID‐19.^[^ ^13^ ^]^ Currently, all of these available vaccines mainly target the S protein of the early strain of SARS‐CoV‐2. With the emergence of several variants of distinct lineages, it is important to ascertain whether these mutations in the S protein in VOCs will affect the immune protection of existing vaccines. In particular, some mutations found in VOCs are located in known antibody‐binding regions.^[^ ^14^ ^]^ Based on the reports regarding mRNA vaccines, mRNA‐1273/BNT162b2 could effectively neutralize pseudoviruses carrying the SARS‐CoV‐2 spike glycoprotein of the Alpha variant. However, the neutralization titers of these sera against the Beta variant decreased by 12.4‐fold for the Moderna mRNA vaccine and 10.3‐fold for the Pfizer mRNA vaccine.^[^ ^15^ ^]^ In addition, the Novavax recombinant protein‐based COVID‐19 vaccine, NVX‐CoV2373, was tested in a Phase 3 trial in the U.K., which demonstrated efficacy of 96.4% against the early strain, 86.3% against the B.1.1.7 (Alpha) variant,^[^ ^13a^ ^]^ while a Phase 2b trial in South Africa demonstrated only 51% efficacy against the B.1.351 (Beta) variant.^[^ ^16^ ^]^ The neutralizing titers of convalescent and vaccine sera are reduced against the variants with these VOCs, as has been reported by us and others.^[^ ^12^, ^17^ ^]^ Interestingly, we recently collected the convalescence sera from patients infected with either the early strain or Delta strain of SARS‐CoV‐2. By comparing the neutralizing antibody (NAb) titers in the convalescent sera against different variants, we observed a significant reduction in the NAbs titer in the Delta convalescence sera against the early strain, and vice versa.^[^ ^18^ ^]^ This “seesaw effect” suggests that different antibody germlines are used for early strain and Delta strain. Taken together, these observations provide important new insight that vaccines specially targeting the Delta variant should be taken into consideration. Strategies for developing updated vaccines to simultaneously cover the early strain, Delta variant and other variants to induce broad‐spectrum neutralizing antibodies are necessary.

The spike proteins of VOCs have been investigated as second‐generation vaccine candidates to tackle the challenges associated with protection against emerging variants of SARS‐CoV‐2.^[^ ^19^ ^]^ Considering the global dominance of the Delta variant, we designed a B.1.617.2(Delta)‐RBD‐nanoparticle (NP) vaccine, which induced potent humoral immune responses and neutralizing antibodies against the novel SARS‐CoV‐2 variants. Meanwhile, bivalent‐RBD‐NP (D614G/B.1.617.2) and trivalent‐RBD‐NP (D614G/B.1.351/B.1.617.2) vaccines were developed to enhance the broad‐spectrum protective effect. Together, these updated vaccines are hoped to prevent immune evasion of dominant the Delta strain, and provide broader coverage over currently circulating strains, including the early strain, Delta, Beta/Gamma, and other variants.

## 2 Results

### 2.1 Construction and Characterization of the B.1.617.2 (Delta)‐RBD Nanoparticle Vaccines

**Figure 1:**
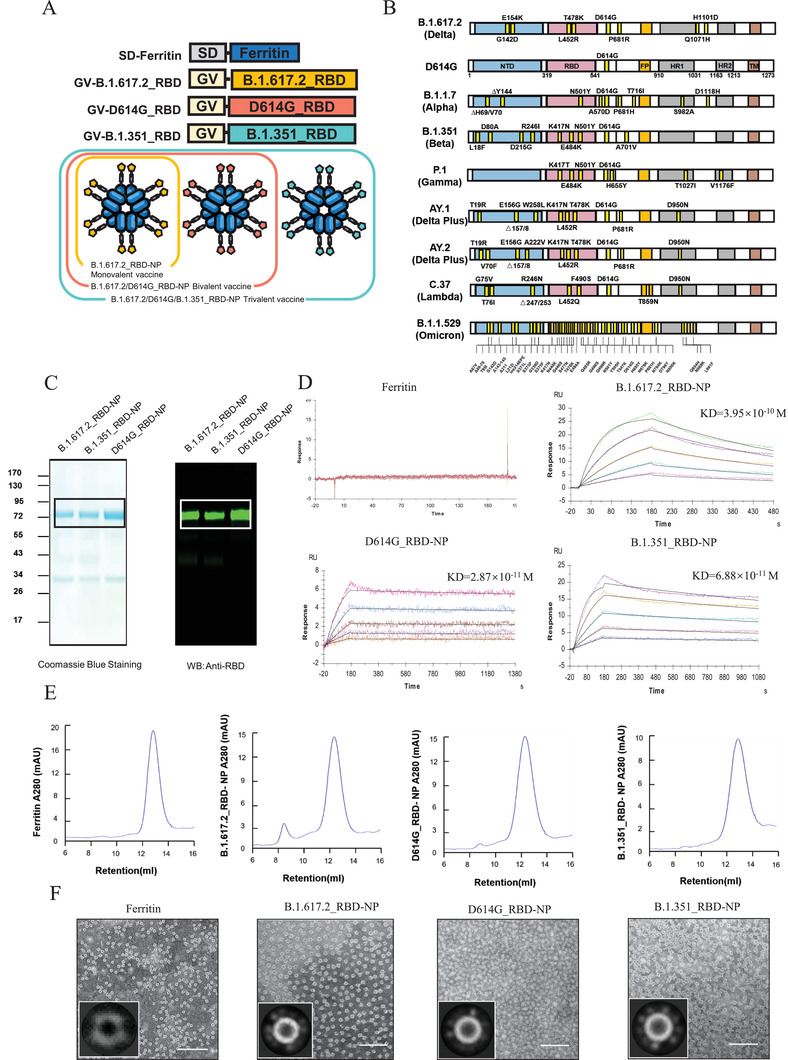
Characterization and purification of the RBD‐NP vaccines A) The schematic of the multiple RBD‐NP vaccines. The bivalent vaccine is composed of Gv‐D614G_RBD‐NP and Gv‐B.1.617.2_RBD‐NP at the ratio of 1:1. The trivalent vaccine is composed of Gv‐D614G_RBD‐NP, Gv‐B.1.351_RDB‐NP, and Gv‐B.1.617.2_RBD‐NP, in which the ratio is 1:1:1 for each. SD: SdCatcher. Gv: GvTagOpti. B) Schematic of mutations in spike protein of SARS‐CoV‐2 variants. C) Coomassie blue staining (left) of B.1.617.2_RBD‐NP, D614G_RBD‐NP, and B.1.351_RBD‐NP. The expression and purity of each protein were confirmed by western blotting with RBD antibodies (right). The bands in the box indicate the pure NPs. D) The binding affinity of each RBD NP to hACE2 protein was analyzed with Surface plasmon resonance (SPR). The ferritin protein was used as a negative control. The displayed KD value shown was the average of three independent experiments. E) The SEC of D614G_RBD NP, B.1.351_RBD‐NP, and B.1.617.2_RBD‐NP. The ultraviolet absorptions at 280 were shown. The retention volume represented the peak of each nanoparticle. F) TEM images and 2D clustering analysis of each RBD‐NP. Samples were negatively stained. Scale bars represented 100 nm.

In view of the significant number of breakthrough infections and morbidity of Delta variant around the world, we developed a B.1.617.2 (Delta) RBD nanoparticle vaccine and adapted the RBD nanoparticle (RBD‐NP) system, which was previously developed by us,^[^ ^20^ ^]^ to target the Delta variant (**Figure** **1**A). Simultaneously, we also developed multivalent nanoparticle vaccines to facilitate the cross‐protection of multiple variants. To overcome the possible immune evasion of early strains to the Delta‐based vaccine, which was evidenced by the significant reduction in the NAbs titer in the Delta convalescence sera against the early strains,^[^ ^18^ ^]^ a bivalent vaccine was generated, containing B.1.617.2_RBD‐NP and D614G (early)_RBD‐NP at a ratio of 1:1. Considering the other reported VOCs, we also chose to display B.1.351 (Beta/Gamma) _RBD to represent the key mutations (K417T/N, E484K, and N501Y) in the RBD region (Figure 1B). Thus, trivalent RBD‐NP vaccines were developed with a mixture of B.1.617.2_RBD‐NP, D614G_RBD‐NP, and B.1.351_RBD‐NP in a ratio of 1:1:1. To connect RBD immunogen and self‐assembled ferritin particles, we adapted a GvTagOpti/SdCatcher (GV/SD) system which derived from *Gardnerella Vaginalis* and *Streptococcus Dysgalactiae* respectively and developed by us.^[^ ^21^ ^]^ The Sd‐coding sequence was genetically fused to the N‐terminus of ferritin (Sd‐Ferritin), while the Gv‐coding sequence was fused at the N‐terminus of B.1.617.2_RBD, D614G_RBD, and the B.1.351_RBD sequence. The purified Gv‐B.1.617.2_RBD, Gv‐D614G_RBD, and Gv‐B.1.351_RBD were irreversibly and covalently conjugated to Sd‐Ferritin to generate B.1.617.2_RBD‐NP, D614G_RBD‐NP and B.1.351_RBD‐NP, followed by purification by gel filtration (Figure 1A). The expression and purity of the corresponding nanoparticle conjugates were verified by Coomassie blue staining and western blotting (Figure 1C). We further determined the antigenicity of RBD‐NPs by detecting their binding affinity and kinetics between RBD‐NPs and human angiotensin‐converting enzyme 2 (hACE2). The measured binding affinity constants (KD) of the B.1.617.2_RBD‐NP, D614G_RBD‐NP, and B.1.351_RBD‐NP with the hACE2 receptor were 3.95 × 10^−10^, 2.87 × 10^−11^, and 6.88 × 10^−11^ M, respectively, indicating that the epitopes on the NPs were correctly exposed and was able to functionally bind with the hACE2 receptors (Figure 1D). The purity and homogeneity of nanoparticles was also verified by size exclusion chromatography (SEC) and transmission electron microscopy (TEM) (Figure 1E,F). After antigen conjugation, RBD‐NP turned to spikes protruding from the spherical core compared with the smooth spherical 24‐mer nanoparticles formed by ferritin only (Figure 1F). All these experiments confirmed the successful production of NPs displaying the properly folded RBD proteins of SARS‐CoV‐2 variants on their surfaces.

### 2.2 The RBD‐NP Vaccine Induces Potent Humoral Immune Responses in BALB/c Mice

**Figure 2:**
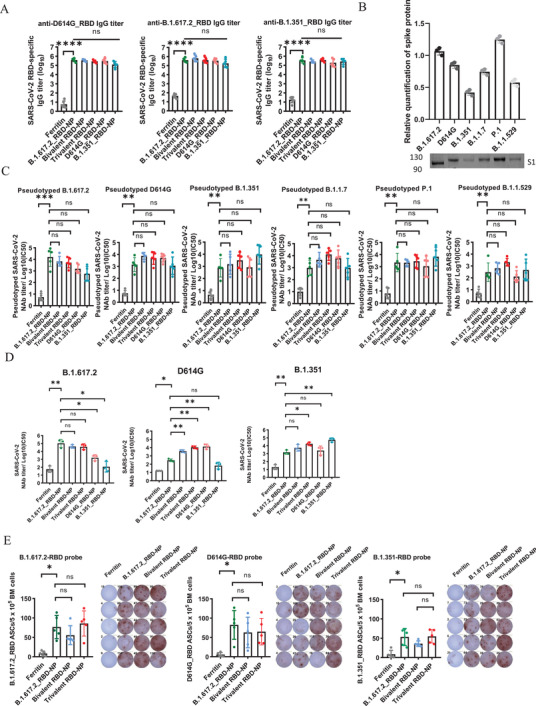
Immune responses in nanoparticles vaccinated BALB/c mice. A) B.1.617.2_RBD‐, D614G_RBD‐, and B.1.351_RBD‐specific IgG titers of immunized BALB/c mice at week 6 were detected by ELISA. IgG antibody titers of serum were determined by serial dilution and the data are represented as the reciprocal of the endpoint serum dilution (*n* = 5). B) The S proteins of these pseudoviruses were detected by western blot and qualified it by Image J. C) Serialized diluted serum was detected to neutralize antibodies against pseudotyped SARS‐CoV‐2 (B.1.617.2, D614G, B.1.1.7, B.1.351, P1, B.1.1.529). The data represented NAbs NT50 within each group. Experiments were conducted independently in triplicates (*n* = 5). D) FRNT50 of NAbs of each group of authentic SARS‐CoV‐2 virus (D614G/B.1.351/B.1.617.2) was determined by the FRNT and represented by half‐maximum inhibitory concentrations (IC50) (*n* = 3). E) Bone marrow (BM) was collected at two weeks postboost vaccination. The quantification of RBD‐specific IgG+ antibody‐secreting cells (ASCs) was determined by ELISPOT (*n* = 5). Data represented as mean ± SD, Brown‐Forsythe and Welch ANOVA with Dunnett's T3 multiple comparisons test was used. *p ≤ 0.05, **p ≤ 0.01, ***p ≤ 0.001, ****p ≤ 0.0001, ns = not significant.

To evaluate the immunogenicity of the above vaccines, BALB/c mice were immunized with a 12 µg dose of B.1.617.2_RBD‐NP adjuvant with Alhydrogel. The mice were also immunized with 6 ug of each B.1.617.2/ D614G _RBD‐NP (Bivalent RBD‐NP) and 4 ug of each B.1.617.2/D614G/B.1.351/_RBD‐NP (Trivalent RBD‐NP), respectively. As a control, mice were immunized with 12 µg of ferritin, D614G_RBD‐NP, B.1.351_RBD‐NP. All mice were boosted with the vaccine at week 4. The sera were collected and the mice were euthanized 2 weeks after the boost. The RBD‐NP vaccines induced RBD‐specific IgG at titers of approximately 10^5^, except in the ferritin negative control group (**Figure** **2**A), which indicated that the RBD‐NP had a strong immunogenicity. To further evaluate the immunogenicity of the above vaccines, we performed pseudovirus neutralization assays as described previously.^[^ ^19^ ^]^ we detected the S proteins of these pseudoviruses by western blot and qualified it by Image J (Figure 2B). The same amount of pseudoviruses was put in according to the quantitative results. The results showed that the NAbs induced by these RBD‐NP vaccines strongly inhibited all of the pseudotyped variants including B.1.617.2, D614G, B.1.1.7, B.1.351, P.1, and B.1.1.529 (Figure 2C). To study whether these sera could inhibit the infection of the authentic B.1.617.2, D614G and B.1.351 strains, a focus reduction neutralizing test (FRNT) was conducted.^[^ ^20^ ^]^ We found that the B.1.617.2_RBD‐NP vaccine significantly inhibited the replication of all the authentic B.1.617.2, D614G, and B.1.351 strains, the FRNT50 reached to 1.04 × 10^5^, 3.01 × 10^2^, or 1.50 × 10^3^, respectively. Notably, the multivalent RBD‐NP vaccine induced a higher robust neutralization titer, with broader cross‐neutralization. The FRNT50 reached to 4.17 × 10^4^, 3.72 × 10^3^, or 5.50 × 10^3^ in mice immunized with bivalent vaccine and 3.8 × 10^4^, 1.07 × 10^4^, or 1.70 × 10^4^ with trivalent vaccine (Figure 2D).

To measure the number of antigen secreting cells (ASCs) induced by the B.1.617.2_RBD‐NP vaccine, we euthanized BALB/c mice immunized with different vaccines at week 6. The bone marrow cells were evaluated for ASCs with Elispot assay.^[^ ^22^ ^]^ We found that these vaccines generated more RBD‐specific ASCs (Figure 2E) than ferritin. Based on the findings above, we concluded that the B.1.617.2_RBD‐NP vaccine induced robust humoral immune responses and that multivalent RBD‐NP vaccines may produce broader cross‐protection than the B.1.617.2_RBD‐NP only vaccine.

### 2.3 B.1.617.2_RBD‐NP Vaccination Protects Mice against B.1.617.2 Challenge

**Figure 3:**
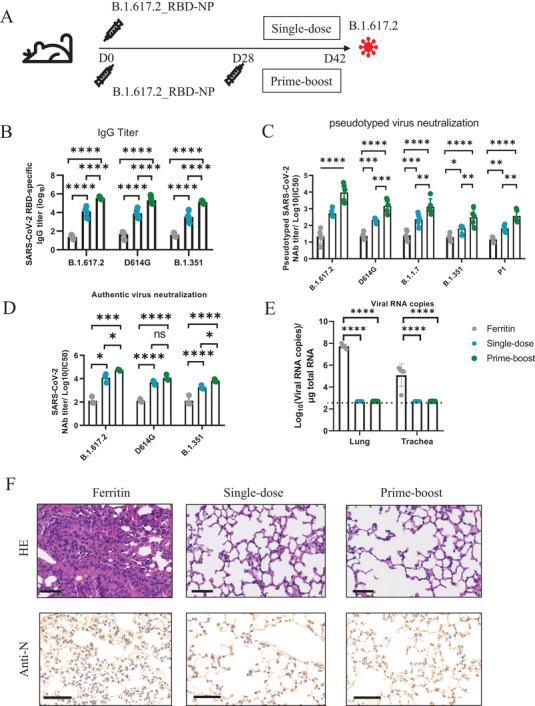
Protection efficacy of B.1.617.2_RBD‐NP vaccine with a prime‐boost or a single‐dose vaccination strategy against SARS‐CoV‐2 variants in hACE2 mice. A) Schematic of hACE2 mice vaccination and challenge. Five mice within each group were vaccinated with B.1.617.2_RBD‐NP vaccine at day 0 or vaccinated with B.1.617.2_RBD‐NP vaccine at day 0 and day 28 by prime‐boost. All mice were challenged with authentic B.1.617.2 SARS‐CoV‐2 at day 42 and were euthanized 3 d postchallenged. B) Serum RBD‐specific IgG antibody assays were determined with ELISA by serum dilution and represented as reciprocal serum dilution at the point of effect (*n* = 5). C) The NAbs titer for SARS‐CoV‐2 pseudovirus of B.1.617.2_RBD‐NP vaccinated hACE2 mice by pseudotyped virus neutralization assay and represented as IC50 (*n* = 5). D) The authentic SARS‐CoV2 virus neutralization antibody NT50 of B.1.617.2_RBD‐NP vaccinated hACE2 mice serum. FRNT50 of NAbs of each vaccine group was determined by FRNT and represented as IC50 (*n* = 3). E) Copies of viral RNA in the lung and trachea of each mouse were identified by qRT‐PCR and plotted in log10 copies per µg total RNA (*n* = 5). The dotted lines indicate the lower detection limit (500 copies mL^−1^) . F) HE staining and IHC against N proteins were evaluated in lungs of each mice. Data represented as mean ± SD, Two‐way ANOVA with Turkey's correction for multiple comparisons was used. *p ≤ 0.05, **p ≤ 0.01, ***p ≤ 0.001, ****p ≤ 0.0001, ns = not significant.

We subsequently immunized hACE2 mice with the B.1.617.2_RBD‐NP vaccine in a single dose or prime‐boost manner to compare the protective effects of different immunization strategies (**Figure** **3**A). In the single‐dose group, hACE2 mice were only immunized with the B.1.617.2_RBD‐NP vaccine. In the prime‐boost group, hACE2 mice were immunized with the B.1.617.2_RBD‐NP vaccine and boosted with the same vaccine on day 28. All the sera were collected on day 42. We found that the mice in both the single dose group and the prime‐boost group generated a high level of RBD‐specific IgG at approximately titers of 10^4^ and 10^5^, respectively, which indicated that the prime‐boost strategy can induce a stronger immune response (Figure 3B). We further conducted pseudovirus neutralization assays to evaluate the NAbs. The NAbs induced by the B.1.617.2_RBD‐NP vaccine strongly inhibited all pseudotyped strains of VOCs (Figure 3C). FRNT assays were conducted to evaluate whether the induced antibodies could neutralize the authentic SARS‐CoV‐2. The neutralizing antibody titers of both vaccine groups, which were represented by FRNT50, were much higher than those of the ferritin group (Figure 3D). The FRNT50 titers of the single dose and the prime‐boost group were 1.17 × 10^4^ and 5.37 × 10^4^ against B.1.617.2, 4.86 × 10^3^ and 1.17 × 10^4^ against D614G, 1.78 × 10^3^ and 6.46 × 10^3^ against B.1.351.

To evaluate the protective activity of B.1.617.2_RBD‐NP against the B.1.617.2 variant, all the mice were challenged with the authentic B.1.617.2 variant with 2 × 10^5^ FFU at day 42, and the mice were euthanized 3 d postchallenge. Viral RNAs in the lungs and tracheas were detected using quantitative RT‐PCR. The control hACE2 mice had an average of 5.25 × 10^7^ and 1.20 × 10^5^ copies per µg total RNA for B.1.617.2 in the lung and trachea respectively (Figure 3E), while the single‐dose and the prime‐boost vaccine‐immunized hACE2 mice had undetectable levels of viral RNA. Histopathological analysis of the lungs indicated that the B.1.617.2 variant challenge induced severe lung lesions similar to the other variants, which were characterized by thickened alveolar septa and the infiltration of inflammatory cells (Figure 3F). Nevertheless, the lungs of mice in the single dose and the prime‐boost groups showed no pathological changes. Immunohistochemical assays against SARS‐CoV‐2 nucleocapsid (N) proteins revealed that the lungs of mice immunized with ferritin were densely distributed with N‐expressing cells, while none was observed in any vaccine‐immunized mice (Figure 3F). All these experiments indicated that the B.1.617.2_RBD‐NP vaccination efficiently protected mice against the B.1.617.2 variant challenge.

### 2.4 Trivalent RBD‐NP Vaccine Exhibits Potent Cross‐Protection against the Variants of SARS‐CoV‐2

**Figure 4:**
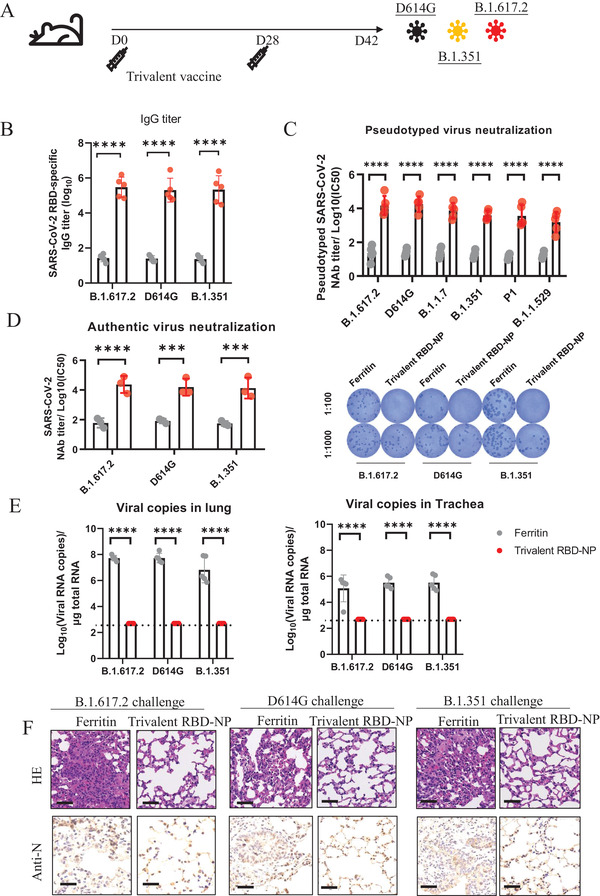
Protection efficacy and breadth of trivalent vaccine against SARS‐CoV‐2 variants in hACE2 mice. A) Schematic of hACE2 mice vaccination. Five mice within each group were prime‐boost vaccinated with trivalent vaccine at day 0 and day 28. All mice were challenged with authentic SARS‐CoV‐2 at day 42 and were euthanized 3 d postchallenged. B) RBD‐specific IgG antibodies titers of serum which were collected at day 28 were determined by serial dilution and represented as the reciprocal of the endpoint serum dilution (*n* = 5). C) The NAbs titer for SARS‐CoV‐2 pseudovirus of vaccinated hACE2 mice by pseudotyped virus neutralization assay and represented as IC50 (*n* = 5). D) The serum of each mouse was tenfold serially diluted and incubated with authentic SARS‐CoV‐2 (B.1.617.2/D614G/B.1.351), followed by incubating with Vero E6 cells. The FRNT spots of each well were counted. FRNT50 of NAbs of each vaccine group was determined by FRNT and represented as IC50 (*n* = 3). E) Viral RNA copies in the lung and trachea of each mouse were determined by qRT‐PCR and plotted as log10 copies per µg total RNA (*n* = 5). The dotted lines indicate the lower detection limit (500 copies mL^−1^). F) HE staining and IHC against N proteins were evaluated in lungs of each mice. Data represented as mean ± SD, Two‐way ANOVA with Turkey's correction for multiple comparisons was used. *p ≤ 0.05, **p ≤ 0.01, ***p ≤ 0.001, ****p ≤ 0.0001, ns = not significant.

To further determine the protective activity of multivalent RBD‐NPs against SARS‐CoV‐2 variants, we immunized hACE2 mice with the trivalent RBD‐NP (D614G/B.1.351/B.1.617.2) vaccine, with a boost administered on day 28 (**Figure** **4**A). We found that the sera of mice at day 42 after immunization contained a high titer of RBD‐specific IgG (Figure 4B). In the pseudovirus neutralization assays, the NAbs induced by trivalent RBD‐NP vaccine exhibited a robust inhibitory effect on a variety of variants, including B.1.617.2, D614G, B.1.1.7, B.1.351, P.1, and B.1.529 pseudotyped variants with the titers of 1.48 × 10^4^, 1.75 × 10^4^, 7.43 × 10^3^, 3.83 × 10^3^, 3.61 × 10^3^, and 2.80 × 10^3^, respectively (Figure 4C). FRNT assays were performed to evaluate NAbs against authentic SARS‐CoV‐2 variants. The data showed that the neutralizing antibodies titers of RBD‐NP vaccine groups against variants, which were represented by FRNT50, were all over 1.0 × 10^4^ (Figure 4D).

To evaluate the protective activity of the trivalent RBD‐NP vaccine against the authentic D614G, B.1.351, and B.1.617.2 variants, mice at week 6 after vaccination were challenged with 2 × 10^5^ FFU of D614G, B.1.351, or B.1.617.2 strain respectively, and the mice were subsequently euthanized 3 d postchallenge. Viral RNAs in the lungs and tracheas were detected using quantitative RT‐PCR. The control hACE2 mice had an average of 5.25 × 10^7^, 5.32 × 10^7^, and 6.67 × 10^6^ copies per µg total RNA in the lung and 1.20 × 10^5^, 3.18 × 10^5^, and 3.28 × 10^5^ copies per µg total RNA in the trachea for the B.1.617.2, D614G, and B.1.351 variant respectively. In contrast, hACE2 mice immunized with trivalent RBD‐NP vaccine had undetectable levels of viral RNA in both the lung and trachea tissues (Figure 4E). Hematoxylin‐eosin staining of the lungs indicated that all the SARS‐CoV‐2 variants induced severe lung lesions, characterized by thickened alveolar septa and infiltration of inflammatory cells (Figure 4F). Nevertheless, the lungs of mice in the trivalent RBD‐NP vaccine groups showed no pathological changes. Immunohistochemical assays against the SARS‐CoV‐2 nucleocapsid (N) proteins revealed that the lungs of mice immunized with ferritin were densely distributed with N‐expressing cells, while none was observed in any vaccine‐immunized mice (Figure 4F). These data demonstrated that the trivalent RBD‐NP vaccination efficiently protected mice against B.1.617.2, D614G, and B.1.351 variants challenges.

### 2.5 The Trivalent RBD‐NP Vaccine Elicits Potent Neutralizing Ab Responses in Rhesus Macaques

**Figure 5:**
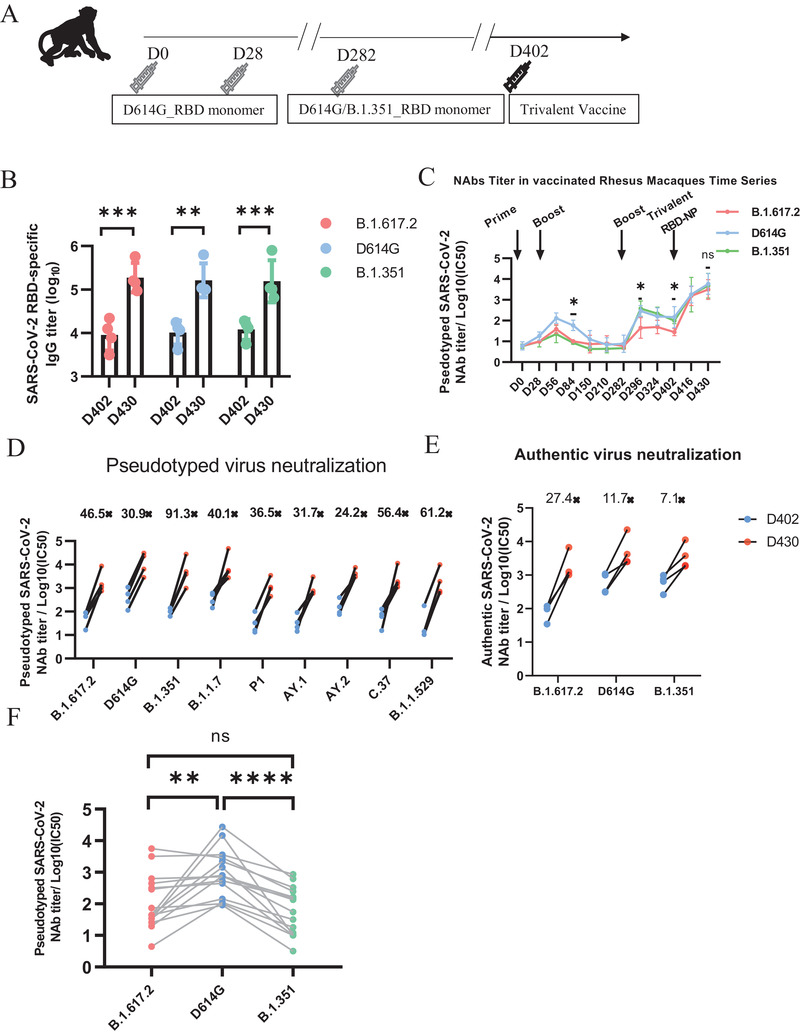
The boost with the trivalent RBD‐NP vaccine in rhesus macaques induces cross‐neutralization of SARS‐CoV‐2 variants. A) Schematic of rhesus macaques vaccination. Four monkeys were immunized with D614G_RBD monomer intramuscularly in a prime‐boost mode and vaccinated at the day 0 and day 28. At day 282, the third dose of D614G/B.1.351_RBD monomer was given an intramuscular injection. At day 402, four monkeys were immunized with the trivalent RBD‐NP vaccine. B) B.1.617.2_RBD, D614G_RBD‐specific and B.1.351_RBD‐specific IgG antibodies titers of Rhesus Macaques serum before and post the fourth dose of trivalent vaccine were determined using ELISA by serial dilution (*n* = 4). C) The NAbs titer for SARS‐CoV‐2 pseudovirus (D614G/B.1.351/B.1.617.2) was determined by FRNT and plotted as a time‐course curve. D) The NAbs titer for SARS‐CoV‐2 pseudovirus (D614G/ B.1.1.7/ B.1.351/ P.1/ B.1.617.2/ AY.1/ AY.2/ C.37/B.1.1.529) of rhesus macaques before and post the fourth dose of trivalent vaccine was determined by pseudotyped virus neutralization assay and represented as IC50. Each dot represents serum from one animal (*n* = 4). E) Fold changes in neutralization against authentic SARS‐CoV‐2 viruses (D614G, B.1.351 and B.1.617.2) from the fourth dose of the trivalent vaccine (*n* = 4). F) The NAbs titer for SARS‐CoV‐2 pseudovirus (D614G/ B.1.351/ B.1.617.2) of convalescent serum was determined by pseudotyped virus neutralization assay and represented as IC50. Each dot represents serum from one patient (*n* = 14). Data represented as mean ± SD, Two‐way ANOVA with Turkey's correction for multiple comparisons was used. *p ≤ 0.05, **p ≤ 0.01, ***p ≤ 0.001, ****p ≤ 0.0001, ns = not significant.

To evaluate whether nanoparticle vaccines induced prophylactic NAbs against SARS‐CoV‐2 variants in nonhuman primates, we immunized rhesus macaques with trivalent RBD‐NPs. These rhesus macaques were immunized with 50 µg doses of D614G_RBD monomer on day 0 and day 28 and followed by 50 µg doses of D614G/B.1.351_RBD monomer on day 282, as we have already confirmed in previous studies that monomer immunization did not induce as many protective NAbs as trivalent RBD‐NPs (**Figure** **5**A). However, RBD‐specific IgG titers against the B.1.617.2, D614G, and B.1.351 strain were quite high at day 28 after the re‐boost with trivalent RBD‐NP vaccines, reaching to 1.87 × 10^5^, 1.63 × 10^5^, and 1.55 × 10^5^ increased by 20.8‐, 15.9‐, and 12.8‐fold (Figure 5B). At the same time, the NAb titers against pseudotyped D614G, B.1.351 and B.1.617.2 virus were also significantly increased (Figure 5C). In addition, we found that the trivalent RBD‐NP vaccine booster could induce NAbs against other SARS‐CoV‐2 variants, including B.1.1.7, B.1.351, P.1, AY.1, AY.2, C.37, and B.1.1.529, at 28 d after vaccination (Figure 5D). In particular, this newer boost enhanced the NAb titer to B.1.617.2 with a 46.5‐fold increase (Figure 5D). Furthermore, the authentic D614G, B.1.351 and B.1.617.2 variants were mixed with serially diluted sera collected at day 28 after the re‐boost with trivalent RBD‐NP vaccines to investigate the protective activity. The data showed that the neutralization titers increased by 11.7‐, 7.1‐, and 27.4‐fold for D614G, B.1.351, and B.1.617.2, respectively (Figure 5E). Fourteen convalescent sera were used as controls for the pseudovirus neutralizing assay against SARS‐CoV‐2 variants (Figure 5F). The convalescent sera were less effective to neutralize B.1.351 and B.1.617, in comparison with its neutralization to D614G strain (Figure 5F). These results indicate that trivalent RBD‐NP vaccines produced effective protective NAbs in rhesus monkeys and significantly prevented the infection of D614G, B.1.351, or B.1.617.2.

## 3 Discussion

Because the genome of SARS‐CoV‐2 constantly undergoes mutation, the development of vaccines against the early strain do not induce efficient cross‐protection.^[^ ^17^, ^23^ ^]^ The emergence of several SARS‐CoV‐2 VOCs has also raised concerns about the increased transmission and potential evasion from vaccine‐induced immunity. There is a significant reduction of cross‐neutralization against SARS‐CoV‐2 between the early strain and the Delta strain in the convalescent sera, this so called “seesaw effect” indicates that different antibody germlines are used to respond to the early strain and Delta strain.^[^ ^18^ ^]^ Thus, a new vaccine strategy to induce the production of a broader spectrum antibodies is necessary. In this study, the monovalent B.1.617.2_RBD‐NP vaccine and trivalent RBD‐NP vaccines has already demonstrated to be an efficient immunogenic strategy against the Delta variant and other multiple variants. This approach supports the rapid adjustment for RBD‐based vaccines, allowing to quick respond to the evolution of SARS‐CoV‐2 viruses. It is notable that, because rhesus macaques are difficult to obtain, we did not use monomer RBD‐NP vaccine as a control in the third boost. Therefore we cannot distinguish whether the immune response was specifically elicited by the trivalent RBD‐NP vaccine or whether it could be elicited by any three‐time boost. In addition, due to the limitations of the experimental platform, we did not challenge the rhesus macaques with authentic viruses. Instead, we challenged the hACE2 mice with authentic viruses to demonstrate the protection efficacy of the trivalent RBD‐NP vaccine. Importantly, we found that the broad neutralizing antibodies against different SARS‐CoV‐2 variants were generated in rhesus macaques after the immunization with the trivalent RBD‐NP vaccine.

The recent explosive spread of the Delta variant in 152 countries and its association with breakthrough infections in vaccinated people prompted us to pay attention to the closely related Delta plus variants, such as Delta‐AY.1, Delta‐AY.2 (https://www.cdc.gov/csels/dls/locs/2021/07‐06‐2021‐lab‐advisory‐SARS‐CoV‐2_Variants_AY_1_and_AY_2_Now_Aggregated_with_Delta_Variant_B_1_617_2.html). Interestingly, these two Delta plus variants harbored three mutations K417N, L452R, and T478K in the RBD region and the trivalent vaccine could induce abundant NAbs and the protect against these Delta plus variants. This result may indicate that since K417N is located in the B.1.351‐RBD region and L452R, T478K is located in the B.1.617.2‐RBD region, and the trivalent vaccine could induce different germlines in the antibody reservoir to produce NAbs for neutralizing variants. Although the spike mutations responsible for the immune escape mechanism of VOCs are not yet fully understood, the RBD has been identified as a major target in the induction of NAbs. As we chose different RBD of VOCs as immunogens, antibodies with different cross‐protection abilities could be induced. Further studies related to the preferential expression of the subset of immunoglobulin heavy chain, the frequency of somatic hypermutation, and the dominant clonal lineages after vaccination could be used to optimize the vaccine design.

Although extensive vaccination has been performed in a large number of countries, the Delta variant has triggered further epidemic worldwide. The possible change in immunization strategies for vaccines has gradually become an urgent question. In our study, we first evaluated the prime‐boost strategy and single‐dose strategy with B.1.617.2_RBD‐NP vaccine, and found that both strategies could produce sufficient NAbs to have the same protective effect in the virus challenge experiment in hACE2 mice. However, we showed that the prime‐boost strategy can stimulate the production of protective neutralizing antibodies. Consistent with mouse vaccination analyses, all four rhesus macaques, which had previously been inoculated with three doses of RBD monomer vaccine and received third boost with the trivalent vaccine, exhibited robust neutralizing responses against many pseudoviruses of SARS‐CoV‐2 variants, including the newly emerging AY.1, AY.2 and C.37 variants.^[^ ^11^, ^24^ ^]^ At the end of November 2021, the Omicron variant triggered a new wave of COVID‐19 pandemic.^[^ ^8^ ^]^ We tested the neutralizing activity of the nanoparticle vaccine‐induced antibodies against pseudoviruses of the Omicron mutant and found that trivalent RBD‐NP vaccines could induce the production of large amounts of neutralizing antibodies against Omicron variant in mice and rhesus macaques. These results suggest that the sequential immunization strategy with trivalent SARS‐CoV‐2 vaccines could be used as a potential method to improve vaccination efficacy and induce the production of broad‐spectrum NAbs. The B.1.617.2_RBD nanoparticle monovalent or the trivalent vaccines not only exerts potent protection efficiency, but also has the advantages of high antibody titer, good stability, convenient preservation, and transportation, it may therefore be a quite good candidate for vaccine development.

## 4 Conclusion

Immune evasion remains one of the most challenging sectors in the vaccine development against COVID‐19. The RBD of variants has been recognized as a targetable region with strong immunogenicity to induce high levels of NAbs against SARS‐CoV‐2. Here, we demonstrate that B.1.617.2_RBD‐targeted nanoparticle vaccines exhibit a strong protection of the SARS‐CoV‐2 Delta variant, as well as the trivalent RBD‐NP exhibit a broad protection of the SARS‐CoV‐2 Delta variant and other variants. When administered prior to virus challenge in the hACE2 mice, B.1.617.2_RBD‐NP vaccines induce humoral immunity to produce NAbs, and subsequently preventing the hACE2 mice from infection by SARS‐CoV‐2 infection. We believe that this novel B.1.617.2_RBD‐NP vaccines could constitute a powerful prophylactic approach for high‐risk people, whose countries are currently under attack by SARS‐CoV‐2 variants.

## 5 Experimental Section

### Ethics Statements

The Ethics Review Committee of Sun Yat‐sen University approved the study. All animal experiments were carried out in compliance with the guidelines and regulations of the Laboratory Monitoring Committee of Guangdong Province of China, and approved by the Ethics Committee of Zhongshan Medical College (ZSSOM) of Sun Yat‐sen University Laboratory Animal Care (Guarantee Number: 2017–061). Authentic SARS‐CoV‐2 challenge studies were approved by the Ethics Committee of ZSSOM of Sun Yat‐sen University on Laboratory Animal Care (Assurance Number: 2017–061) as well. Nonhuman primates experiments were approved by the Institutional Animal Care and Use Committee (IACUC) of Guangdong Landau Biotechnology Co, Ltd. (IACUC Approval No: LDACU 20200216‐01). Convalescent sera of COVID‐19 patients were obtained from Guangzhou 8th People's Hospital and Fifth Affiliated Hospital of Sun Yat‐sen University. The Ethics Review Boards of Sun Yat‐sen University, Guangzhou 8th People's Hospital approved this study(Guarantee Number: 201803040002). All the participants were given written informed consent with approval of the Ethics Committees.

### Cells and Viruses

HEK293T, CHO‐K1, and Vero E6 cells were obtained from ATCC. These adherent cells were cultured in DMEM supplemented with 1% penicillin‐streptomycin (ThermoFisher) and 10% FBS (ThermoFisher). HEK293T expressing hACE2 (hACE2/HEK293T) was constructed in home. All cells were tested for mycoplasma DNA using PCR and confirmed that they were not contaminated by mycoplasma.

The authentic SARS‐CoV‐2 (hCoV‐19/CHN/SYSU‐IHV/2020 strain; accession number on GISAID: EPI_ISL_444969), GDPCC‐nCOV84 (accession number on National Genomics Data Center: NPRC2.062100001), and GDPCC‐nCoV‐Delta related experiments were carried out in the BSL‐3 facility of Sun Yatsen University.

### Animal Models

Transgenic hACE2 mice (C57BL/6) were purchased from GemPharmatech Co, Ltd. The generation procedure was described as published before.^[^ ^20^ ^]^ Specific‐pathogen‐free (SPF) 5–6 week female BALB/c mice were purchased from Guangdong Medical Laboratory Animal Center. All mice were housed and vaccinated in SPF facilities at Laboratory Animal Center of Sun Yat‐sen University. Four adult rhesus macaques (2 male, 2 female) between 2 and 4 year old were purchased previously from Guangdong Landau Biotechnology Co, Ltd. Monkeys experiments were conducted according to the guidelines and regulations of Laboratory Monitoring Committee of Guangdong Province of China.

### Quantitative Reverse Transcription Polymerase Chain Reaction (qRT‐PCR)

Lung and Trachea of challenged hACE2 mice were collected and homogenized with gentleMACS M tubes (Miltenyi Biotec, 130‐093‐236) in a gentle‐MACS dissociator (Miltenyi Biotec, 130‐093‐235). RNAs were extracted using RNeasy Mini Kit (QIAGEN, 74104) according to the manufacturer's instruction, followed by the qRT‐PCR to determine the viral RNA copies of different tissues utilizing one‐step SARS‐CoV‐2 RNA detection kit (PCR‐Fluorescence Probing) (Da An Gene Co., DA0931). To generate a standard curve, the SARS‐CoV‐2 nucleocapsid (N) gene was cloned into a pcDNA3.1 expression plasmid for standards. The indicated copies of N standards were tenfold serially diluted from 10^9^ to 10^3^ and proceeded to qRT‐PCR utilizing the same one‐step SARS‐CoV‐2 RNA detection kit to obtain standard curves. The reactions were carried out on a QuantStudio 7 Flex System (Applied Biosystems) according to the manufacturer's instruction. The viral RNA copies of each tissue were calculated into copies per µg total RNA and presented as log10 scale.

### SARS‐CoV‐2 Infection

Specific‐pathogen‐free (SPF), transgenic hACE2 mice (C57BL/6), which have been immunized with different vaccines, were challenged with authentic SARS‐CoV‐2 in BSL‐3 facility. SARS‐CoV‐2 strains, named as hCoV‐19/CHN/SYSU‐IHV/2020 (Accession ID on GISAID: EPI_ISL_444969), GDPCC‐nCoV‐84 and GDPCC‐nCoV‐Delta representing D614G, B.1.351 and B.1.617.2, respectively, were used to challenge mice. Mice were anaesthetized with isoflurane and inoculated intranasally with 2 × 10^5^ FFU of SARS‐CoV‐2 viruses. The lungs were collected at 3 d postinfection (d.p.i.).

### Histopathology and Immunohistochemistry

The hACE2 mice challenged with SARS‐CoV‐2 were euthanized in BSL‐3 facility. Lungs were collected and fixed in 4% paraformaldehyde buffer for 48 h, followed by embedding with paraffin. Longitudinal sections were performed on these tissues. The sections (3–4 µm) were stained with hematoxylin and eosin (H&E). For immunohistochemistry, lung sections of each mice were incubated with rabbit anti‐SARS‐CoV‐2 Nucleoprotein (N) at 1:200 dilution and the IHC were conducted as published before.^[^ ^20^ ^]^

### Protein Expression and Purification

The construction of RBD nanoparticle vaccine was as described above.^[^ ^20^ ^]^ To further improve the binding efficiency of the NP vaccine, the Gv/Sd connection system was used to enhance the efficiency and production of the SARS‐CoV‐2 NP vaccine. Sd‐Ferritin was expressed and purified from E. coli.^[^ ^21^ ^]^ Briefly, the Sd gene was fused to the N‐terminus of the Ferritin gene, and the DNA sequence of Sd‐Ferritin was cloned into the pET28a vector. The constructed plasmid was transformed into BL21 (Takara) prokaryotic expression bacteria. A single clone was picked and amplified by shaking in LB containing kanamycin at 37 °C. When the OD value of the bacterial solution reached 0.4–0.6 ratio, isopropyl b‐D‐1 thiogalactopyranoside (IPTG) (Takara) was added to the bacterial solution to induce protein expression. 18 h after induction, the bacteria expressing the protein were harvested and lysed by ultrasound. After centrifugation, the supernatant was loaded on a Sepharose 6 FF (GE Healthcare) size exclusion column, pre‐equilibrated with 20 × 10^−3^ m Tris 50 × 10^−3^ m NaCl buffer (pH 7.5), and the protein was eluted at the same buffer under the rate of 10 mL min^−1^. The total column volume (Vt) of Sepharose 6 FF was 53 mL, and the elution volume (Ve) of Sd‐ferritin NP was 26 mL. The concentration of Sd‐ferritin was determined by the BCA assay method. The bacterial endotoxins in nanoparticles were quantified by the Tachypleus amebocyte lysate test (≤10 EU per dose). Afterward, Coomassie blue staining, size‐exclusion chromatography (SEC), and transmission electron microscopy (TEM) were used to confirm purity and homogeneity.^[^ ^25^ ^]^

D614G_RBD, B.1.351_RBD, and B.1.617.2_RBD were expressed and purified from Chinese Hamster Ovary K (CHO‐K1) cells. Briefly, the Gv was genetically fused at the N‐terminus of the D614G_RBD, B.1.351_RBD, and B.1.617.2_RBD at the downstream of secretory signal peptide (SP), respectively. The DNA sequences of SP‐Gv‐D614G_RBD, SP‐Gv‐B.1.351_RBD, and SP‐Gv‐B.1.617.2_RBD were codon‐optimized for mammalian cells and cloned into the vector pLVX plasmid. HEK293T cells were co‐transfected with the constructed pLVX plasmid, the psPAX2 (Addgene) plasmid, and PLP/VSVG by using polyethyleneimine (PEI, Sigma) cultured in DMEM medium. The recombinant viruses in the supernatant were collected 60 h after transfection and then infected with anchorage‐dependent CHO‐K1 cells cultured in an F12K medium. After 24 h, the F12K medium was replaced with CHO S4 medium for cell suspension and expansion to a density of 3 × 10^6^ cells mL^−1^. After 7 d, the supernatant was collected by centrifugation. The clarified supernatant passes through the filter (Repligen) fitted with the filtration system KR2i Tangential Flow with the 10 and 100 kDa molecular weight cutoff (MWCO) to obtain the molecular weight protein from 10 to 100 kDa. The obtained concentrate was purified by AKTA pure system purification (GE Healthcare) through a Capto SP Impres column at a flow rate of 5 mL min^−1^ and eluted with 150 × 10^−3^ m NaCl phosphate buffer (PH = 7) buffer. The harvested protein was replaced by a regular Tris buffer. Protein concentration was detected by BCA measurement, and Coomassie blue staining was performed to confirm purity.

### Western Blot

In brief, pseudovirus were collected and lysed with the lysis buffer (150 × 10^−3^ m NaCl, 50 × 10^−3^ m Tris–HCl [pH 7.5], 1 × 10^−3^ m EDTA,1% Triton X‐100, 0.5% NP‐40), plus PMSF and protease inhibitor cocktail for 30 min at 4˚C. The lysates were clarified by centrifugation at 12 000 g for 10 min at 4 °C. The samples were analyzed by SDS‐PAGE and detected by western blotting. Image J was used to quantify the western blotting results. SARS‐CoV‐2 Spike Antibody (40592‐MM117, SinoBiological) was used to detected the spike protein.

### Size‐Exclusion Chromatography (SEC)

Gv‐D614G_RBD, Gv‐B.1.351_RBD and Gv‐B.1.617.2_RBD were incubated with Sd‐Ferritin in the Tris‐HCl buffer. After 8 h, the bound protein was subjected to Superose 6 Increase 10/300 (GE Healthcare) size exclusion column that was pre‐equilibrated with 20 × 10^−3^ m Tris 50 × 10^−3^ m NaCl buffer (pH 7.5) at a rate of 0.8 mL min^−1^. D614G_RBD‐NP, B.1.351_RBD‐NP andB.1.617.2_RBD‐NP were eluted in retention of retaining 11–14 mL.

### Surface Plasmon Resonance (SPR)

The recombinant hACE2 protein was immobilized on a CM5 Sensor Chip (carboxymethylated dextran covalently attached to a gold surface) with the amine coupling kit (GE Healthcare). Ferritin‐only nanoparticles, D614G_RBD‐NP, B.1.351_RBD‐NP, and B.1.617.2_RBD‐NP were preincubated with serial concentrations, including 4 × 10^−9^, 2 × 10^−9^, 1 × 10^−9^, 0.5 × 10^−9^, and 0.25 × 10^−9^ m in the HEPS buffer and then injected (30 µL min^−1^), respectively. The signals were recorded by the Biacore T100 instrument with the standard protocols.

### Animal Vaccination

In terms of immunization of BALB/c mice, there are five BALB/c mice in each group. In trivalent vaccine group mice, each mouse used 4 µg of D614G_RBD‐NP, 4 µg of B.1.351_RBD‐NP, and 4 µg of B.1.617.2 RBD‐NP to form a trivalent nanoparticle vaccine and an equal volume of Alum (InvivoGen) Adjuvant emulsification for subcutaneous immunization. The D614G/B.1.617.2_RBD‐NP mice were vaccinated subcutaneously with 6 µg of D614G_RBD‐NP and 6 µg of B.1.351_RBD‐NP. Mice from the D614G_RBD‐NP, B.1.351_RBD‐NP, and B.1.617.2_RBD‐NP groups were subcutaneously immunized with a 12 µg monovalent vaccine. The mice of the ferritin group were immunized with equimolar ferritin, which was the same as the trivalent vaccination group. All mice were prime/boost vaccinated with NP vaccines at week 0 and week 4. Serum was collected every two weeks. The mice were euthanized in the sixth week.

For hACE2 mouse vaccination, all mice were vaccinated according to BALB/c mice; for a single‐dose strategy, the mice received an authentic SARS‐CoV‐2 challenge six weeks after vaccination and were euthanized 3 d after the challenge. The sera were collected every two weeks. For prime‐boost strategy, the mice received a real SARS‐CoV‐2 challenge two weeks after the booster vaccination and were euthanized 3 d after the challenge. The sera were collected every two weeks.

For the rhesus monkey vaccination, four monkeys were immunized with 50 µg D614G_RBD monomer intramuscularly in a prime‐boost mode and vaccinated in the 0th and 4th weeks. Monkey serum was taken once every two weeks. On day 282, a 50 ug D614G/B.1.351_RBD monomer was given an intramuscular injection. On day 402, four monkeys were immunized with 50 µg trivalent vaccine. Serum was collected every two weeks after the fourth dose of immunization. All vaccines were formulated with an equal volume of Alum (InvivoGen) adjuvant.

### Enzyme Linked Immunosorbent Assay (ELISA)

Recombinant proteins D614G_RBD, B.1.351_RBD, and B.1.617.2_RBD were diluted at one level at 5 µg mL^−1^ concentration in coating buffer to be coated on 96 high‐bond well plates (Corning) overnight at 4 °C, respectively. After being washed three times with PBS, the plates were blocked with 5% nonfat milk/PBS for 1 h. After another series of washings, the immunized animal serum was serially diluted, added to each well in duplicate, and then incubated at room temperature for 1 hr. The detection of antigen‐specific IgG antibody in serum of BALB/c mice, hACE2 mice, or rhesus macaques was conducted through adding HRP‐conjugated goat antimouse or goat antimonkey secondary antibody (Invitrogen) respectively at dilution of 1:10 000 and incubating for another 1 h after washing with PBS/T (containing 1% Tween‐20) three times. The plates were washed four times before adding 100 µL of TMB (eBioscience) solution at each well. After 5 min of chromogenic progress at room temperature, a 100 µL stop solution (Solarbio) was added to quench the reaction followed by an absorption measure at 450 nm. The data were analyzed using GraphPad Prism 8.0 software for nonlinear regression to calculate endpoint titers.

### Focus Reduction Neutralizing Test (FRNT)

Briefly, the Vero E6 cells were plated in 96‐well plates at a density of 2 × 10^4^ cells per well and incubated until the cells reached a confluence of 90–100%. The sera from BALB/c mice, hACE2 mice, and rhesus macaques were 10 times serially diluted. Five hundred FFU of genuine SARS‐CoV‐2 viruses were mixed with diluted serum at a ratio of 1:1 to 1 h incubation time at 37 °C. The cell culture medium was removed from the 96‐well plate and incubated with a virus/serum mixture.

The plates were subsequently incubated for 1 h at 37 °C. DMEM containing 1.6% CMC was added to each incubated well for 24 h following removal of the supernatant. The following day, the supernatant was released, and the cells were secured with 200 µL of 4% paraformaldehyde in each well. After incubating again at 4 °C for 12 h, the fixative was removed and the plates were washed with 200 µL of PBS for 3 times. Then 100 µL of PBS containing 0.2% Triton X‐100 and 1% BSA were added per well. After reaction for 30 min at room temperature, each well was washed with 200 µL PBS for 3 times. Primary antibody (Anti‐SARS‐N; 40143‐T62‐100) was diluted to 1:1000 with PBS solution containing 1% BSA. 50µL of the diluted primary antibody was add to every well and incubated at 37 °C for 1 h. After the primary antibodies were incubated, the cell of each well was washed 3 times with 200 µL of PBS/T (0.1% Tween). Secondary antibody (goat bunny HRP IgG; SSA004‐1) was diluted at 1:2000 with a 1% BSA solution of PBS. 50 µL of the dilute secondary antibody was added to each well and incubated at 37 °C for 1 h, then washed three times with PBS/T. 50 µL TrueBlue (KPL) was added to every well and adjusted for 5 min shaking at room temperature. The plates were washed with ddH_2_O, and the liquid was eliminated, followed by point counting using the ImmunoSpot microanalyzer. The reduction rates of the serial dilution assay were analyzed by GraphPad Prism 8.0 using nonlinear regression to measure the FRNT50 titer.

### Pseudotyped Virus Neutralization Assay

The generation protocol was described as published before.^[^ ^17a^ ^]^ Briefly, HEK293T cells were co‐transfected with the psPAX2 (Addgene) plasmid, the lentiviral plasmid expressing luciferase (Addgene), and the plasmid expressing the respective mutant spike proteins by using polyethyleneimine (PEI, Sigma). 48 h after transfection, the culture supernatant was collected and filtered with a 0.20 µm filter, and then stored at −80 °C. Virus titration was performed by serially diluting the viral infection of hACE2‐293T cells, and the infectivity was measured by detecting luminescence. The serum of all immunized animals was serially diluted and incubated with a pre‐titrated amount of pseudotyped SARS‐CoV‐2 virus at 37 °C for 1 h. Subsequently, the serum/virus mixture was added to the wells containing 2 × 10^4^ hACE2‐293T cells and incubated at 37 °C in 5% CO_2_ for 48 h. Then the cells were lysed with lysis buffer (Promega), and the lysate was measured by detecting the relative luminescence unit (RLU) in the photometer (Promega) to measure the luciferase activity. GraphPad Prism 8.0 software was used to analyze the serum neutralizing antibody titers of the pseudotyped virus.

### Elispot

A detailed protocol was previously published.^[^ ^22^ ^]^ Briefly, multiScreen HTS IP 879 filter plates of 0.45 µm (Millipore Sigma, MSIPS4W10) were coated with 2.5 µg mL^−1^ SARS CoV‐2 D614G RBD, B.1.351 RBD or B.1.617.2 RBD in coating buffer overnight at 4 °C, respectively. Plates were washed three times with PBS and blocked with complete DMEM for at least 1 h at 37 °C. The single‐cell suspensions of BM cells were plated in plates at a density of 5 × 10^5^ cells per well. Antibody‐secreting cells (ASC) of BALB/c mice BM cells were detected by ALL‐IN‐ONE mouse ELISPOT Accessory kit (Dakewe) according to the manufacturer's protocol. Antigen‐specific spots were then calculated by ImmunoSpot software (Cellular Technology Ltd.).

### Statistical Analysis

The statistical details of the specific experiment, including the statistical test used, number of samples, mean values, standard error of the mean (SD) and p values, were described in the figure legends. For comparison between each group with the mean of every other group within a dataset containing more than two groups, Brown‐Forsythe and Welch ANOVA with Dunnett's T3 multiple comparisons test was used. Two‐way ANOVA with Turkey's correction for multiple comparisons was used when comparing the mean of more than two groups against a single baseline or control group. *p ≤ 0.05, **p ≤ 0.01, ***p ≤ 0.001, ****p ≤ 0.0001, ns = not significant. Statistical analyses were conducted utilizing Graphpad Prism 8.0.

## Conflict of Interest

The authors declare no conflict of interest.

## Data Availability

The data that support the findings of this study are available in the supplementary material of this article.
